# Oxidative Stress in *Caenorhabditis elegans*: Protective Effects of Spartin

**DOI:** 10.1371/journal.pone.0130455

**Published:** 2015-06-26

**Authors:** Timothy Truong, Zachary A. Karlinski, Christopher O’Hara, Maleen Cabe, Hongkyun Kim, Joanna C. Bakowska

**Affiliations:** 1 Department of Molecular Pharmacology and Therapeutics, Loyola University Chicago, Maywood, Illinois, United States of America; 2 Department of Cell Biology and Anatomy, Rosalind Franklin University of Medicine and Science, North Chicago, Illinois, United States of America; CSIR-Central Drug Research Institute, INDIA

## Abstract

Troyer syndrome is caused by a mutation in the *SPG20* gene, which results in complete loss of expression of the protein spartin. We generated a genetic model of Troyer syndrome in worms to explore the locomotor consequences of a null mutation of the *Caenorhabditis elegans SPG20* orthologue, F57B10.9, also known as *spg-20*. *Spg-20 *mutants showed decreased length, crawling speed, and thrashing frequency, and had a shorter lifespan than wild-type animals. These results suggest an age-dependent decline in motor function in mutant animals. The drug paraquat was used to induce oxidative stress for 4 days in the animals. We measured survival rate and examined locomotion by measuring crawling speed and thrashing frequency. After 4 days of paraquat exposure, 77% of wild-type animals survived, but only 38% of *spg-20* mutant animals survived. Conversely, animals overexpressing *spg-20* had a survival rate of 95%. We also tested lifespan after a 1 hour exposure to sodium azide. After a 24 hour recovery period, 87% of wild type animals survived, 57% of *spg-20* mutant animals survived, and 82% of animals overexpressing *spg-20* survived. In the behavioral assays, *spg-20 *mutant animals showed a significant decrease in both crawling speed and thrashing frequency compared with wild-type animals. Importantly, the locomotor phenotype for both crawling and thrashing was rescued in animals overexpressing *spg-20*. The animals overexpressing *spg-20* had crawling speeds and thrashing frequencies similar to those of wild-type animals. These data suggest that the protein F57B10.9/SPG-20 might have a protective role against oxidative stress.

## Introduction

Hereditary spastic paraplegias are a group of inherited neurological disorders characterized by progressive muscle weakness and spasticity of the lower extremities [1–3]. Hereditary spastic paraplegias are typically categorized into two groups. The first group is considered “pure” and exhibits only two symptoms, lower-extremity spasticity and paraparesis. The second group is considered “complicated” because additional non-neurological symptoms are present [4]. Troyer syndrome is a complicated hereditary spastic paraplegia that manifests as spasticity of the lower limbs as well as cognitive disability, dysarthria, and short stature [5]. The disease is caused by an autosomal recessive frameshift mutation on the *SPG20* gene, which results in the complete loss of expression of the spartin protein, suggesting that the pathogenesis involves a loss-of-function process [6].

Spartin is a multifunctional protein that harbors three conserved domains. A microtubule-interacting and -trafficking domain (MIT) and a plant-related senescence domain (PSD) are found at the N-terminus and C-terminus, respectively [7]. Recently, we also identified and characterized the ubiquitin binding region (UBR) [8]. MIT is known to function in cytokinesis, and PSD is involved in mitochondrial physiology [9]. We showed that the UBR in spartin binds to Lys-63–linked ubiquitin chains and is important for the occurrence of aggresome-like induced structures [8]. The presence of these structural domains in spartin, along with its interaction with multiple binding partners [10–11] and its association with membranes of several cellular organelles, including endosomes, lipid droplets, and mitochondria [12–14] indicates that the spartin protein plays diverse roles in the biology of the cell and on an organismal level.

*Caenorhabditis elegans* has been used as a model for many neurodegenerative diseases, including Parkinson’s, Huntington’s, and amyotrophic lateral sclerosis [15–16]. We turned to the nematode *C*. *elegans* to examine whether spartin affects motor behavior or influences the oxidative stress response. In this study, we focused on the orthologue of SPG20 in *C*. *elegans*, classified as F57B10.9, also named *spg-20*. We then characterized it by using several behavioral assays. We observed that *spg-20(tm5514)* mutants had a shortened lifespan and a decrease in motor function. In addition, *spg-20(tm5514)* mutants showed hypersensitivity to oxidative stress, whereas overexpression of spg-20 resulted in recovery of resistance to oxidative stress. These data suggest that spartin has a protective effect within the oxidative stress response.

## Materials and Methods

### Microinjection and transformation

Animals with the deletion allele *tm5514* were obtained from the National Bioresource Project (Tokyo, Japan). *spg-20(tm5514)* was twice backcrossed to wild-type N2 animals before further experiments were performed. To generate transgenic lines that carry an extrachromosomal array, a mixture of DNA constructs, including a fosmid and a co-injection marker, was prepared at a final concentration of 100 ng/μl by adjusting the concentration with pBluescript and injected into the gonads of wild-type or *spg-20(tm5514)* animals. Specifically, *spg-20(tm5514)* animals were injected with a rescue fosmid (WRM0619aE01) at 10 ng/μl, along with a pan-neuronal co-injection marker H_2_0::GFP at 5 ng/μl. As a control, wild-type N2 animals were injected with H_2_0::GFP at 5 ng/μl. The transgenic line exhibiting the highest transmission rate of the co-injection marker among the resulting transgenic lines was chosen for further crossbreeding and analysis.

### Amino acid sequence alignment

The F57B10.9 protein sequence in *C*. *elegans* (http://www.ncbi.nlm.nih.gov/protein/25144380?report=fasta) was aligned with SPG20 in *H*. *sapiens* (http://www.ncbi.nlm.nih.gov/protein/214830079?report=fasta) using BLASTP (http://blast.st-va.ncbi.nlm.nih.gov/Blast.cgi?PROGRAM=blastp&PAGE_TYPE=BlastSearch&LINK_LOC=blasthome). Sequence alignment was done using Clustal omega (http://www.ebi.ac.uk/Tools/msa/clustalo/).

### Lifespan Assay

Age-synchronized animals were established by moving 10–15 gravid adults onto nematode growth medium (NGM, 2.0% bacto-agar, 50mM NaCl, 0.25% bacto-peptone, 1mM CaCl, 5ug/mL cholesterol, 25mM KPO_4_, 1mM MgSO_4_) plates seeded with *Escherichia coli* (OP50) as a source of food for 2 hours. The adult animals were then removed, and eggs were allowed to hatch and grow to larval stage 4 (L4). Synchronized L4 animals were transferred to seeded NGM plates on Day 0. The animals were transferred to fresh NGM plates with food every 2 days for the first 7 days. Animals were then transferred every week thereafter as necessary to maintain an abundant food source. Live and dead animals were counted every day. Animals were counted as dead if they did not respond to repeated prodding with a platinum wire. Animals were excluded from analysis if they exhibited traumatic death by internally hatched progeny or extruded gonads. The assay was carried out at 20°C. Survival curves were generated and analyzed using the Kaplan-Meier method.

### PCR

For PCR studies, 5–10 animals were collected into 50 μl of lysis buffer (50 mM KCl, 10 mM Tris-HCl pH 8.3, 2.5 mM MgCl_2_, 0.45% Triton X-100, 0.45% Tween-20, 10 μl of 20mg/mL proteinase K in 1mL). Samples were frozen for 30 minutes at -80°C. Samples were then incubated at 60°C overnight. The next morning, samples were heated to 95°C for 20 minutes to inactivate proteinase K. DNA lysate (1 μl) was used for PCR amplification using the following primers: *spg-20* common forward (primer B) 5’-GGCAACACCAGTGATTCCGCCTCCAAG-3’, *tm5514* reverse (primer C) 5’-CAGTCGCTTAGCGCCGGGATTTCGAA-3’, and *spg-20* wild-type reverse (primer A) 5’-GACTCCAGTGCTTCGTAACGAATTCGGA-3’.

### Oxidative stress resistance assays

For the oxidative stress assays, paraquat (N,N’-dimethyl-4,4’-bipyridinium dichloride) (Sigma-Aldrich, St. Louis, MO) was used to induce stress. L4 animals were placed on NGM plates containing 2 mM paraquat with OP50 as a source of food. The assay was carried out at 20°C, and the number of living animals was counted every 24 hours for 4 days. Animals were counted as dead if they did not respond to repeated prodding with a platinum wire. The assay was repeated for a total of 4 trials with at least 25 animals per trial. Survival curves were generated and analyzed using the Kaplan-Meier statistical method.

Additionally, we used sodium azide (NaN_3_) to confirm the effects of oxidative stress on the animals. L4 stage animals were placed on fresh NGM plates containing 100mM sodium azide for 1 hour. The assay was carried out at 20°C. The animals were then washed twice in M9 buffer, and placed on NGM plates with OP50 as a food source and kept at room temperature overnight. Survival was measured after 24 hours. Animals were counted as dead if they did not respond to repeated prodding with a platinum wire. The assay was repeated for 5 trials with at least 100 animals per test group. Survival was analyzed using the Kaplan-Meier statistical method.

### Measurement of average crawling speed

The speed of crawling animals was measured on a fresh NGM plate. Day 1-stage adult animals were moved onto an NGM plate without food and allowed to acclimate for 10 seconds. Digital videos of animal movement were acquired using an Olympus SZX7 dissecting microscope (Center Valley, PA) equipped with a 3.2 Megapixel OLYMPUS Q-Color3 digital camera (Melville, NY) and Q-Capture Pro 7 imaging software. The videos were recorded at 1 x 1 binning for 500 frames at 20 frames per second. The videos were analyzed using the open-source wrMTrck plugin for ImageJ software available publicly on the internet (http://www.phage.dk/plugins/wrmtrck.html). At least 30 animals per genotype were analyzed.

### Measurement of thrashing frequency

Thrashing frequency was measured while the animals were suspended in fluid. Day 1-stage adult animals were moved onto a fresh NGM plate containing M9 buffer. Digital videos of animal movement were acquired using a Zeiss Discovery.V8 dissecting microscope (Dublin, CA) equipped with a Canon EOS Rebel T3i camera (Melville, NY). The videos were recorded for 30 seconds at 25 frames per second. The thrashing frequency of the animals, measured in body bends per second, was quantified using the open-source wrMTrck plugin for ImageJ software (http://www.phage.dk/plugins/wrmtrck.html). One body bend was defined as a change in direction of bending at the midbody [17]. At least 30 animals per genotype were analyzed.

## Results

### Generation of *spg-20* mutants in *C*. *elegans*

**Fig 1:**
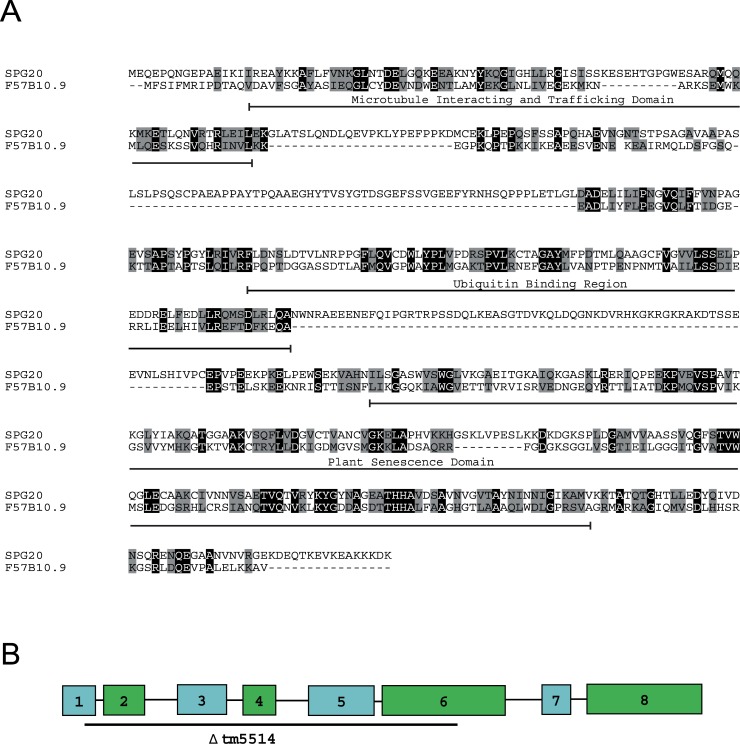
Protein F57B10.9 in *C*. *elegans* is the orthologue of SPG20 in *H*. *sapiens*. (A) Protein sequence alignment using Clustal omega of F57B10.9 and SPG20. These sequences share 23% identity and 65% similarity. Conserved amino acids are marked in grey, and identical amino acids are marked in black. (B) F57B10.9 has one predicted transcript, and the *tm5514* deletion mutation spans exons 1 through 6.

In *H*. *sapiens*, the *SPG20* gene that codes for spartin protein has a mutation that leads to loss of function and neurodegeneration of the cortical spinal projections. To better understand the pathogenesis and the possible behavioral changes resulting from the loss of spartin, we used the model organism, *C*. *elegans*, and characterized the *C*. *elegans* ortholog of *SPG20* in *H*. *sapiens*, the F57B10.9 gene, also known as *spg-20*. We examined the *tm5514* deletion, which results in a null allele of *spg-20*. The spartin protein in *C*. *elegans* shares 23% identity and 65% similarity with SPG20 in *H*. *sapiens* (Blast e-value 3x10^-23^). Spartin contains three evolutionarily conserved domains, including the MIT, the UBR, and the PSD [7–8]. MIT spans amino acids 14–90 in F57B10.9 (a protein product of its cognate gene) and 16–95 in SPG20 and shares 20% identity and 62% similarity. UBR spans amino acids 270–367 in SPG20 and shares 29% identity and 60% similarity between F57B10.9 and SPG20. PSD spans amino acids 261–439 in F57B10.9 and 427–613 in SPG20 and shares 25% identity and 77% similarity (Fig 1A).

**Fig 2:**
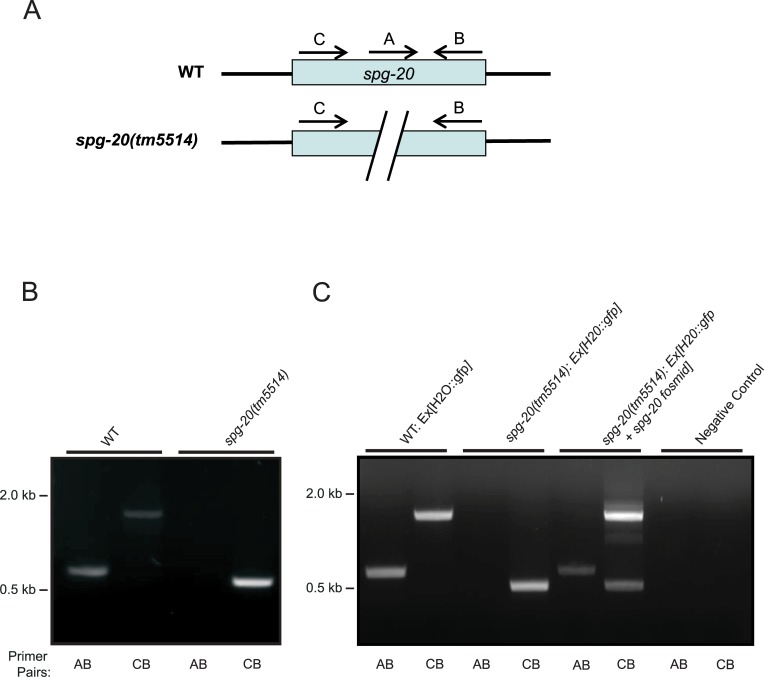
Deletion of *spg-20* in transgenic animals. (A) Schematic diagram shows primers annealing on both WT and *spg-20* mutant genes. (B) Deletion of *spg-20* was confirmed by PCR genotyping. Two sets of primers were used to confirm complete deletion of *spg-20* in mutant strains. Primer pairs AB amplified the wild-type (WT) genome (600 bp), and primer pairs CB amplified the mutant genome (500 bp). (C) PCR confirms deletion of *spg-20* as well as presence of *spg-20* containing plasmid in transgenic animals expressing GFP. The presence of a band at 600 bp using primer pairs A and B and a band at 1700 bp using primers C and B are indicative of wild-type animals. A 500 bp band using primer pairs C and B along with the absence of a band using primer pairs A and B are indicative of the animals containing the *spg-20(tm5514)* knockout mutation. The presence of a band at 600 bp using primer pairs A and B, along with both a 500 bp and a 1700 bp band using primer pairs C and B is indicative of animals overexpressing *spg-20*, which contain both the *spg-20(tm5514)* knockout mutation and the *spg-20* fosmid.

The *spg-20* gene in *C*. *elegans* contains 8 exons, and the *spg-20(tm5514)* mutation spans exons 1 through 6 (Fig 1B). To confirm the mutation in the *spg-20(tm5514)* animals, we performed PCR using two sets of primers. Primers A and B were used to amplify the wild-type genome (600 bp), and primers C and B were used to amplify the both the wild type and the *spg-20(tm5514)* mutant genomes, which are distinguished by the length of the fragment (500 bp and 1700 bp, respectively) (Fig 2A). Fig 2B illustrates the annealing of the first and second primer sets onto the respective wild-type and mutant genomes. When DNA from wild-type animals was used, we observed a band at approximately 600 bp with primers A and B and a band at approximately 1700 bp with primers C and B, as predicted. When using *spg-20* mutant DNA, we observed no band with primers A and B and a band at approximately 500 bp with primers C and B, as predicted (Fig 2B). The absence of a band with primers A and B and the presence of a smaller band with primers C and B confirmed the deletion of *spg-20* in the mutants.

To further investigate the protective effects of *spg-20* under oxidative stress, we generated three strains of animals expressing GFP, as described in Materials and Methods. We generated GFP animals overexpressing *spg-20*, as well as wild-type and *spg-20(tm5514)* mutant animals, both expressing GFP as a control. Transgenic strains were confirmed using PCR and by GFP expression (Fig 2). Using DNA from wild-type GFP animals, we detected an approximately 600 bp band using primers A and B, as well as a band of approximately 1700 bp with primers C and B. These results are consistent with the wild-type genome and conservation of the *spg-20* gene. Using DNA from *spg-20*(tm5514)-GFP animals, we detected an approximately 500 bp band with primers C and B and no bands using primers A and B. This is consistent with the complete loss of the *spg-20* gene associated with the *spg-20(tm5514)* mutation. Using DNA from the GFP mutants overexpressing *spg-20*, we detected a band at approximately 500 bp using primers C and B, which confirms the presence of the *spg-20(tm5514)* knockout mutation. Importantly, we also detected a band at approximately 600 bp with primers A and B, and a band at approximately 1700bp with the primers C and B, which confirm the presence of *spg-20* containing plasmid and the rescue genotype (Fig 2C).

### Behavioral phenotypes and lifespan differences in *spg-20* mutants

**Fig 3:**
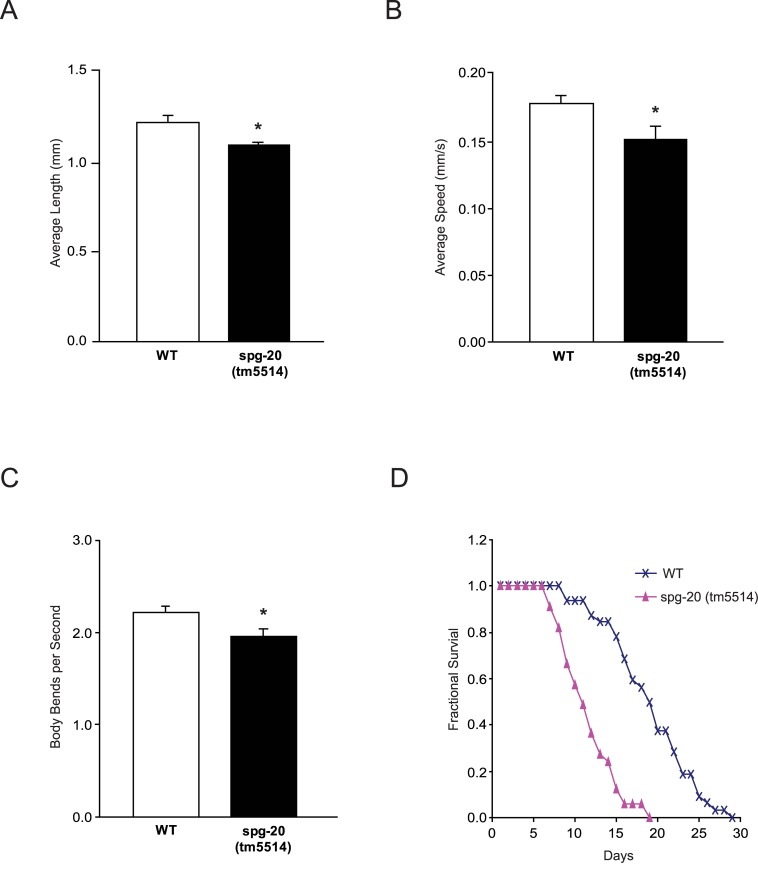
*spg-20* mutant animals exhibited reduced locomotor function and shortened lifespan. (A) Day 1-stage adults were used to measure average length of wild-type (WT) and *spg-20(tm5514)* mutant animals. *spg-20(tm5514)* mutant animals had diminished growth compared with WT animals (*p<0.05). Error bars represent SEM. Two-tailed Student *t* test; n > 30. (B) Day 1-stage adults were used to measure average speed of WT and *spg-20(tm5514)* mutant animals on NGM plates without food. s*pg-20(tm5514)* mutant animals had reduced speed compared with WT animals (*p<0.05). Speed was quantified by video analysis, as described in Materials and Methods. Error bars represent SEM. Two-tailed Student *t* test; n > 30. (C) Day 1-stage adults were used to measure the thrashing frequency (body bends per second) of WT and *spg-20(tm5514*) mutant animals in M9 liquid via video analysis, as described in Materials and Methods. *spg-20(tm5514)* mutant animals had a reduced thrashing rate compared with WT animals (*p<0.05). Error bars represent SEM. Two-tailed Student *t* test; n > 30. (D) Fractional survival of WT and *spg-20(tm5514*) mutant animals. *spg-20(tm5514*) mutant animals had a shorter lifespan than WT animals (p<0.0001). The L4 stage was set as Day 0, and the animals were maintained on OP50 at 20°C for 29 days. Error bars represent SEM. Kaplan-Meier method; n > 30.

To identify differences in phenotypes between s*pg-20(tm5514)* mutant animals and wild-type animals, we measured the length of Day 1-stage adults. Wild-type animals grew to an average length of 1.22 mm (± 0.036 SEM), and s*pg-20(tm5514)* mutants grew to an average length of 1.10 mm (± 0.016 SEM). On average, s*pg-20(tm5514)* mutant animals had diminished length compared with wild-type animals (p<0.05) (Fig 3A). Representative images can be found in S1 Fig. To determine whether s*pg-20* mutants lose locomotor function, we quantified the average speed and thrashing frequency of wild-type and s*pg-20(tm5514)* animals. Videos of individual Day 1-stage adults were recorded and coded for both crawling movement across an agar surface and swimming motion in liquid. The videos were analyzed using the open-source wrMTrck plugin for ImageJ. Wild-type animals displayed an average speed of 0.18 mm/s (±0.0067 SEM) on agar, whereas s*pg-20(tm5514)* animals displayed an average speed of 0.15 mm/s (±0.0096 SEM), a 15% decrease in speed compared to wild-type (p<0.05) (Fig 3B). The frequency of lateral swimming movement in liquid, also known as thrashing, is another conventional method used to characterize locomotor function in *C*. *elegans*. We conducted an automated assessment of the thrashing rate for wild-type and s*pg-20(tm5514)* mutant animals. Wild-type animals displayed an average of 2.23 body bends per second (±0.060 SEM), whereas s*pg-20(tm5514)* mutant animals displayed an average of 1.96 body bends per second (±0.084 SEM). Overall, mutant animals exhibited 10% fewer body bends per second than wild-type animals (p<0.05) (Fig 3C). Because the reduced locomotor function might be indicative of aging [18], we decided to measure the lifespan of wild-type and s*pg-20* mutant animals. Animals did not exhibit differences in survival until after the sixth day. All wild-type animals survived until the eighth day, but animals with the s*pg-20(tm5514)* mutation began dying after the sixth day. Wild-type animals survived up to 29 days, but s*pg-20(tm5514)* mutant animals survived only up to 19 days (p<0.0001) (Fig 3D). The survival data were analyzed using the Kaplan-Meier method, which revealed significant differences between the lifespan of *spg-20* mutant and wild-type animals.

In order to confirm the effects on length and average speed of the *spg-20(tm5514)* mutation on the animals, we used three strains of *C*. *elegans*; wild-type, *spg-20(tm5514)* mutants, and animals overexpressing *spg-20*, all of which expressed green fluorescence protein (GFP). Wild type animals expressing GFP grew to an average length of 1.576 mm (± 0.0321 SEM). *The spg-20(tm5514)* mutant GFP animals grew to an average length of 1.322 mm (± 0.0282 SEM). GFP animals overexpressing spartin grew to an average length of 1.552 mm (±0.0396 SEM). The *spg-20(tm5514)* mutant animals had diminished length when compared to both the wild type animals (p<0.001) and animals overexpressing spartin (p< 0.001). There was no statistical difference found between the average lengths of the wild type GFP animals and the animals overexpressing spartin (S2 Fig).

### Spartin is required for resistance to oxidative stress

We used *C*. *elegans* as a model to investigate the role of spartin in the oxidative stress response. To gain a better understanding of the role of *spg-20* in oxidative stress, we exposed wild-type and s*pg-20(tm5514)* mutant animals to paraquat. Paraquat is readily reduced by an electron donor, such as NADPH, prior to oxidation by an electron receptor, such as dioxygen. These redox reactions produce the reactive oxygen species superoxide (O_2_^-^), which induces oxidative stress [19]. In our oxidative stress assays, wild-type and s*pg-20(tm5514)* mutant animals were placed onto 2 mM paraquat plates for 4 days. This dose has been shown to induce oxidative stress in *C*. *elegans* [20]. The number of animals that were dead or alive was determined each day. Animals with the *spg-20(tm5514)* mutation had a lower survival rate than wild-type animals, indicating higher sensitivity to paraquat (S1 Table). These data suggest that spg-20 has a protective effect in oxidative stress signaling.

**Fig 4:**
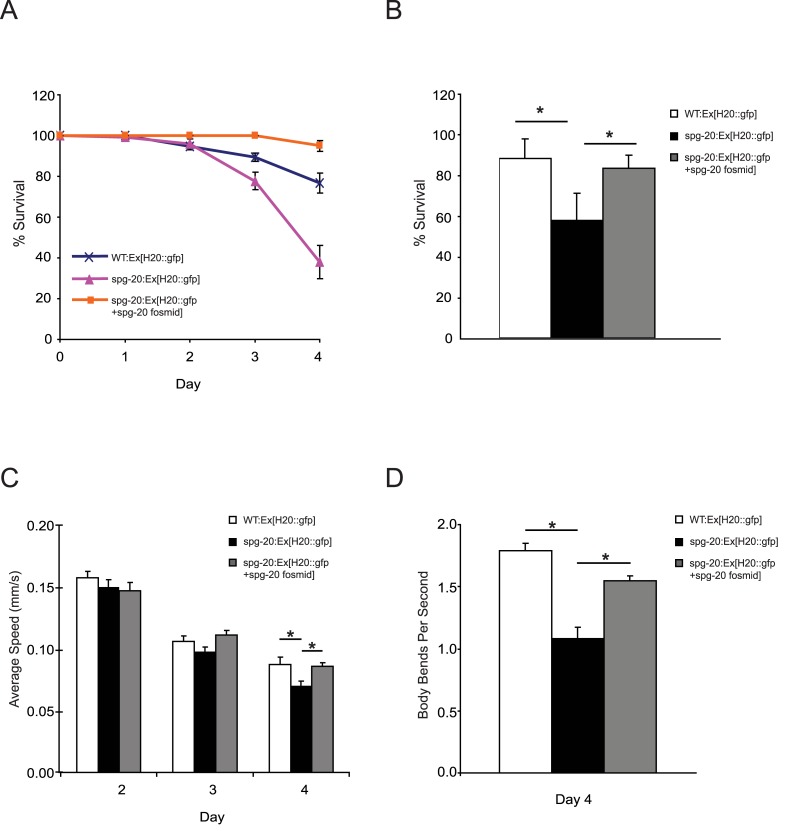
*spg-20* mutant animals are sensitive to oxidative stress. *C*. *elegans* transgenic animals expressing GFP were grown on 2 mM paraquat plates for 4 days. The L4 stage was set as Day 0. (A) Percent survival of animals after treatment with 2 mM paraquat. The percentage of animals alive was scored every 24 hours for 4 days. Animals overexpressing spg-20 had a higher survival rate than both wild-type and *spg-20* mutant animals (*p<0.0001). Survival data represent at least 100 animals per genotype from four independent trials. Error bars represent SEM; Kaplan-Meier method. (B) Percent survival of animals after treatment with 100 mM sodium azide for 1 hour. The percentage of animals alive was scored after a 24 hour recovery period. S*pg-20(tm5514)* mutant animals had decreased survival when compared to wild type animals (*p<0.001) and animals overexpressing spartin (*p< 0.001). There was no significant difference found between the survival of wild type animals and animals overexpressing spartin. (C) Average speed of transgenic animals measured each day for 4 days after treatment with 2 mM paraquat. *spg-20* mutant animals showed reduced speed, whereas animals overexpressing spg-20 showed similar speed compared with WT animals (*p<0.05). Speed was quantified by video analysis, as described in Materials and Methods. Error bars represent SEM. One-way ANOVA with Tukey test; n > 50. (D) Thrashing frequency (body bends per second) of transgenic animals in M9 liquid on Day 4 of treatment with 2 mM paraquat. *Spg-20* mutant animals had a reduced thrashing rate, whereas animals overexpressing spg-20 showed a recovery in thrashing rate compared to WT animals (*p<0.01). Thrashing frequency was measured by video analysis, as described in Materials and Methods. Error bars represent SEM. One-way ANOVA with Tukey test; n > 30.

To further investigate the protective effects of *spg-20* under oxidative stress, we examined three strains of *C*. *elegans*; wild-type, *spg-20(tm5514)* mutants, and animals overexpressing *spg-20*, all of which expressed green fluorescence protein (GFP). All three of these strains were exposed to 2 mM paraquat for 4 days. We observed that s*pg-20* mutant animals had a lower survival rate than wild-type animals (p<0.0001), and animals overexpressing spg-20 had a higher survival rate than both wild-type animals (p<0.001) and s*pg-20* mutant animals (p<0.0001) (Fig 4A). After 4 days of exposure to paraquat, we determined that 77% of wild-type animals survived, 38% of s*pg-20* mutant animals survived, and 95% of animals overexpressing *spg-20* survived (S1 Table). Because animals overexpressing spartin showed significantly longer survival than wild-type animals, we propose that spartin plays an important role in the oxidative stress response.

We studied the effects of an additional oxidative stressor, sodium azide (NaN_3_) which induces oxidative stress by inhibiting cytochrome c oxidase and the electron transport chain [21]. We used three strains of *c*. *elegans*: wild-type, *spg-20(tm5514)* mutants, and animals overexpressing *spg-20*, all of which expressed green fluorescence protein (GFP), and exposed them to 100 mM sodium azide for 1 hour, after which we allowed them to recover on NGM plates seeded with OP50. We measured survival after 24 hours, and found that there was a significant decrease in survival of the *spg-20(tm5514)* when compared to both the wild type animals (p< 0.001) and animals overexpressing spartin (p<0.001) and we found no significant difference when comparing the wild type animals and animals overexpressing spartin (Fig 4B). After the recovery period, 87% of wild type animals survived, 57% of *spg-20* mutant animals survived, and 82% of animals overexpressing *spg-20* survived (S2 Table).

To visualize the effects of paraquat on locomotor function, we measured the average speed and thrashing frequency. No statistically significant differences were observed among the three strains after Day 2 or Day 3 of exposure to paraquat. However, on Day 4 of the crawling assay, wild-type animals had an average speed of 0.087 mm/s (±0.0057 SEM), s*pg-20* mutant animals had an average speed of 0.070 mm/s (±0.0036 SEM), and mutant animals overexpressing *spg-20* had an average speed of 0.086 mm/s (±0.0034 SEM). On average, s*pg-20* mutant animals had a 20% slower average speed than wild-type animals after 4 days of exposure to paraquat (p<0.05). Overexpression of spg-20 in *spg-20* mutant animals led to a recovery in crawling phenotype after 4 days. Specifically, animals overexpressing spg-20 had a 20% higher average speed than s*pg-20* mutant animals (p<0.05). In addition, wild-type animals and animals overexpressing *spg-20* were not statistically different (Fig 4C).

In the thrashing assays, wild-type animals displayed an average of 1.79 body bends per second (±0.057 SEM), s*pg-20* mutant animals displayed an average of 1.19 body bends per second (±0.090 SEM), and mutant animals overexpressing *spg-20* displayed an average of 1.55 body bends per second (±0.039 SEM) (Fig 4D). Overall, s*pg-20* mutant animals showed a 35% lower thrashing rate after the fourth day of exposure to paraquat compared to wild-type animals (p<0.01). In contrast, overexpression of spg-20 resulted in the recovery of thrashing frequency. Animals overexpressing spg-20 had a 25% higher thrashing rate after the fourth day of exposure to paraquat than s*pg-20* mutant animals (p<0.01). Wild-type animals and animals overexpressing spg-20 were not statistically different (Fig 4D). These results suggest that *spg-20* has a protective effect against oxidative stress and provides resistance to a decline in locomotor function.

## Discussion

We examined the behavioral and physical phenotypes of spartin knockout in *C*. *elegans* in comparison to those of wild-type animals. Similar to *Drosophila* [22], mouse [23], and human counterparts, worms with spartin knockout exhibited a marked degree of pathology. Specifically, spartin knockout worms had a slower crawling speed and thrashing frequency, significantly shorter lifespan, and a reduced capacity to cope with oxidative stress.

We identified the F57B10.9 gene in *C*. *elegans* as a spartin ortholog. Spartin protein has high sequence homology and similarity to human spartin in three conserved domains—the MIT domain, the UBR, and the PSD. Of the three conserved domains, the PSD contains the highest level of similarity (25% identity and 77% similarity). The high degree of homology across different phyla indicates that this domain has an important, biological function. Although the exact role of this domain is not known, the PSD has been shown to bind to cardiolipin, a phospholipid that is found in the mitochondrial membrane [9].

We compared *C*. *elegans* spartin knockout mutants to wild-type animals across several parameters. The animals with null expression of spartin exhibited reduced crawling speed and thrashing frequency compared with wild-type animals. This is consistent with studies in mice and in *Drosophila* that show defects in motor function after spartin knockdown [22]. Studies also show that *Drosophila* with spartin knockout had significantly impaired climbing ability; they climb a shorter distance than their wild-type counterparts over the same time span. This also agrees with findings demonstrating that spartin knockout mice have a reduced maximal speed compared with wild-type animals [23].

In this study we also observed that animals with null expression of spartin had a shorter lifespan than the wild-type animals. Not only did the spartin knockout animals start dying sooner, but they also had a significantly shorter maximal lifespan compared with wild-type animals. Several factors could contribute to a reduced lifespan, such as exposure to high temperature, ultraviolet irradiation, increased levels of reactive oxygen species, or pathogens [24]. It is possible that oxidative stress played a role in the decreased lifespan of the mutant animals, as it has been shown to accelerate aging and contribute to neurodegeneration [25].

We next tested the role of spartin in the presence of oxidative stress by comparing spartin knockout animals with wild-type animals and animals overexpressing spartin. After exposing the animals to paraquat (an inducer of oxidative stress) for 4 days, we observed that the mutant animals had a much lower survival rate than the wild-type animals. The animals with overexpressed spartin survived significantly longer than the wild-type animals. Motility was also shown to be affected by oxidative stress [26]. The spartin mutant animals exhibited drastically reduced crawling and thrashing than wild-type controls. In the presence of oxidative stress, animals overexpressing spartin showed a rescue phenotype, as they did for lifespan. These two findings indicate that spartin might be an important factor in protecting against oxidative stress.

The mechanism for the protective function that spartin has on oxidative stress is currently unknown. We speculate that it is related to energy production in the mitochondria, because it has been shown that spartin plays a role in maintaining mitochondrial membrane potential. Specifically, a depletion of spartin causes the mitochondrial membrane to depolarize [9]. Reactive oxygen species, the levels of which paraquat increases, also depolarize the mitochondrial membrane [27]. These two mechanisms could work together to collapse the mitochondrial membrane potential, which would further decrease ATP production and eventually cause the organism to die. This mechanism is consistent with the finding that overexpression of spartin rescues these animals; the overexpression of spartin could help maintain the mitochondrial membrane potential and allow the cell to continue producing ATP, even in the presence of elevated levels of oxidative stress.

*C*. *elegans* has been studied extensively as an animal model for neurodegenerative diseases, such as Parkinson’s disease, amyotrophic lateral sclerosis, and hereditary spastic paraplegia type 4. Two specific stressors that lead to neurodegeneration and have been studied in *C*. *elegans* are osmotic stress in amyotrophic lateral sclerosis [14] and oxidative stress in Parkinson’s disease [16]. In models of Parkinson’s disease, evidence indicates that the protein DJ-1 plays a role in oxidative stress in *C*. *elegans*. Loss of this protein has been shown to lead to mitochondrial fragmentation and accumulation of autophagy markers [28]. Several other proteins were implicated in oxidative stress—induced Parkinson’s disease, including pink-1 and LRK-1. Mutations of these proteins cause increased sensitivity to paraquat and shorted mitochondrial cristae, as well as defects in certain axonal outgrowths [20]. In this study we showed that worms depleted of spartin had a shortened lifespan and a decrease in motor function. In addition, *spg-20(tm5514)* mutants showed hypersensitivity to oxidative stress, whereas overexpression of spg-20 resulted in recovery of resistance to oxidative stress. These data indicate that spartin has a protective effect within the oxidative stress response. Future experiments will examine the mitochondrial morphology and molecular pathway of oxidative stress in worms depleted of spartin and overexpressing spartin.

## Supporting Information

S1 Fig Effect of *spg-20* expression on length of C. elegans. Images of a day 1 L4-stage adult wild-type animal (left) and of a day 1 L4-stage adult *spg-20(tm5514)* mutant animal (right). Representative individuals are shown. All images were taken at the same magnification and treated identically. (Scale Bar: 1 millimeter).
(EPS)

S2 Fig *spg-20(tm5514)* length phenotype is rescued by overexpression of spartin. Day 1-stage adults were used to measure average length of wild-type (WT), *spg-20(tm5514)* mutant animals, and animals overexpressing spartin, all of which expressed GFP. *spg-20(tm5514)* mutant animals had diminished growth compared with WT animals (*p<0.001) and animals overexpressing spartin (*p<0.001). No statistical difference was found between wild type animals and animals overexpressing spartin. Error bars represent SEM. One-way ANOVA with Tukey test; n > 20.
(EPS)

S1 Table Survival of *c*. *elegans* after treatment with paraquat. Mutant animals experience shorter lifespan when compared to wild type animals and animals overexpressing spartin.
(PDF)

S2 Table Survival of *c*. *elegans* after treatment with sodium azide. Animals with the *spg-20(tm5514)* mutation have decreased survival after exposure to sodium azide when compared to wild type animals and animals overexpressing spartin.
(PDF)
