# Supplementary material for: Oxidative Stress in Caenorhabditis elegans: Protective Effects of Spartin
Source: PLoS One. 2015 Jun 26;10(6):e0130455. doi: 10.1371/journal.pone.0130455 (PMC4482654; doi:10.1371/journal.pone.0130455)
Supplement: S1 Table — Mutant animals experience shorter lifespan when compared to wild type animals and animals overexpressing spartin. (PDF) [file pone.0130455.s003.pdf]

Supplementary Table 1

Strains	Median Survival	P value (Kaplan-Meier)	Figure no.	No. of death observed/no. of total animals
WT	19	Reference	3D	32/32
spg-20(tm5514)	11	<0.0001		33/33
WT: Ex[H20::gfp]	n/a	Reference	4A	14/112
spg-20(tm5514): Ex[H20::gfp]	n/a	<0.0001		48/121
spg-20(tm5514): Ex[H20::gfp + spg-20 fosmid]	n/a	<0.001		4/78
