# Supplementary material for: Oxidative Stress in Caenorhabditis elegans: Protective Effects of Spartin
Source: PLoS One. 2015 Jun 26;10(6):e0130455. doi: 10.1371/journal.pone.0130455 (PMC4482654; doi:10.1371/journal.pone.0130455)
Supplement: S2 Table — Animals with the spg-20(tm5514) mutation have decreased survival after exposure to sodium azide when compared to wild type animals and animals overexpressing spartin. (PDF) [file pone.0130455.s004.pdf]

Supplementary Table 2

Strains	Median Survival	P value (Kaplan-Meier)	Figure no.	No. of death observed/no. of total animals
WT: Ex[H20::gfp]	n/a	<0.000000001	4B	21/161
spg-20(tm5514): Ex[H20::gfp]	n/a	Reference		85/198
spg-20(tm5514): Ex[H20::gfp + spg-20 fosmid]	n/a	<0.0001		21/116
